# Health state utilities of patients with hepatitis B and C and hepatitis-related conditions in Japan

**DOI:** 10.1038/s41598-022-21470-3

**Published:** 2022-10-13

**Authors:** Hiroki Sugimori, Maki Hirao, Ataru Igarashi, Hiroshi Yatsuhashi, Shunya Ikeda, Naohiko Masaki, Hiroshi Yotsuyanagi, Takeshi Yoda, Takeshi Odajima, Tomoyuki Takura, Tomohiro Hirao

**Affiliations:** 1 Department of Nursing, Faculty of Sports and Health Science, Daito Bunka University, 560 Iwadono, Higashimatsuyama City, Saitama 3558501 Japan; 2 Division of Hematology, Department of Medicine, Saiseikai Central Hospital, Tokyo, Japan; 3 Unit of Public Health and Preventive Medicine, School of Medicine Medical Course, Yokohama City University, Kanagawa, Japan; 4 National Hospital Organization (NHO) Nagasaki Medical Center, Nagasaki, Japan; 5 School of Medicine, International University of Health and Welfare, Chiba, Japan; 6 National Sanatorium Tamazenshoen, Tokyo, Japan; 7 Division of Infectious Diseases, The Institute of Medical Science, The University of Tokyo, Tokyo, Japan; 8 Department of Public Health, Kawasaki Medical School, Okayama, Japan; 9 Japanese Red Cross Kanto-Koshinetsu Block Blood Center, Tokyo, Japan; 10 Department of Healthcare Economics and Health Policy, Graduate School of Medicine, The University of Tokyo, Tokyo, Japan; 11 Department of Public Health, Faculty of Medicine, Kagawa University, Kagawa, Japan

**Keywords:** Hepatology, Health care economics, Quality of life

## Abstract

Health state utilities are global measurements of quality of life and have been used to evaluate health outcomes for the cost-utility analysis. This study aimed to estimate the health state utilities of patients with hepatitis B (HB), hepatitis C (HC), and hepatitis-related diseases in Japan. We distributed a self-administered questionnaire, including the EuroQol 5-Dimension 5-Level (EQ-5D-5L), to 9,952 outpatients with several clinical conditions caused by HB or HC virus infection (such as asymptomatic chronic hepatitis, chronic hepatitis, compensated cirrhosis, and decompensated cirrhosis) and estimated the condition-specific utilities of patients with HB or HC. In patients with more severe conditions (patients with acute hepatitis, fulminant hepatitis, and hepatocellular carcinoma and patients undergoing post-liver transplantation), the utilities of these severe conditions were estimated by three hepatitis experts using the EQ-5D-5L. The means of the utilities for acute hepatitis, fulminant hepatitis, asymptomatic chronic hepatitis, chronic hepatitis, compensated cirrhosis, compensated cirrhosis, hepatocellular carcinoma stage I/II, hepatocellular carcinoma stage III/IV, and post-liver transplantation were 0.529, − 0.111, 0.904, 0.868, 0.845, 0.722, 0,675, 0,428, and 0.651 and 0.876, 0.821, 0.737, 0.671, 0.675, 0.428, and 0.651 in HB and HC, respectively. To the best of our knowledge, this is the first study that comprehensively assessed the health state utilities of patients with HB, HC and hepatitis-related conditions from a nationwide survey in Japan using the EQ-5D-5L.

## Introduction

Viral hepatitis is the most common blood-borne infection worldwide, causing hepatitis, hepatic cirrhosis, and hepatocellular carcinoma (HCC)^1–3^. In Japan, hepatitis B and C are the largest infectious diseases in our country in Japan. For an example, approximately 3% of people are infected with hepatitis C virus (HCV) infection, resulting in more than 30,000 liver-related deaths annually^4–6^. Although most patients with chronic hepatitis (CH) are asymptomatic in the early phases of the disease^7,8^, the disease can steadily progress to crucial symptomatic life-threatening liver conditions such as cirrhosis and liver cancer^9–12^. Therefore, measures to prevent viral hepatitis are urgently needed. Guidelines for vaccination, viral testing, and treatment of hepatitis B (HB) and hepatitis C (HC) have been developed, and the Hepatitis Treatment Strategy Council consisting of experts in hepatitis developed the "7-year Strategy for Hepatitis Research" in June 2008 and has been promoting research based on this strategy^7–9^.

Moreover, several studies have reported on health-related quality of life (HRQoL) in patients with HB and HC^10,11^. However, in Japan, the assessment of hepatitis treatment, such as the Health Technology Assessment (HTA), could not provide sufficient information on the utilities of each HB- and HC-related condition and economic outcomes of their treatments. Although, this information is necessary for health economic evaluation, it has been suggested that each country should develop its own utilities because some studies have found that there are differences between countries in the observed quality weights for cultural, racial, and ethnic reasons^12^. Therefore, in this study, we first estimated the utility values for each condition of HB and HC for patients receiving standard treatment on a nationwide scale.

A quality-adjusted life year (QALY) is a measure of disease burden, calculated as follows: 1 year of life is multiplied by a utility factor, where a utility of 0 represents death and a utility of 1 represents perfect health. In principle, the utility can be negative when the quality of life is judged to be worse than being dead. Using QALYs, we assume that health or health improvement can be measured based on the amount of time spent in various health conditions and make a decision on treatment. Thus, QALY is necessary for a cost-effectiveness analysis^13^.

There are many methods for calculating the utilities. The National Institute for Health and Care Excellence (NICE) guidelines in the UK, which produce evidence-based guidelines for health, public health, and social care practitioners, recommend that the EuroQol 5-Dimension (EQ-5D) is the preferred measure of HRQoL in adults. The EQ-5D is universally used and has been validated in many populations, and it is composed of five dimensions of health: mobility, self-care ability, ability to undertake usual activities, pain and discomfort, and anxiety and depression. The system has been designed in such a way that people can describe their own HRQoL^14^. However, there are few or no such studies using a new descriptive system, EuroQol 5-Dimension 5-Level (EQ‑5D‑5L), with five response levels (no problems, slight problems, moderate problems, severe problems, and extreme problems) in patients with hepatitis in Japan.

Our study aimed to estimate the health state utilities of HB, HC, and hepatitis-related liver diseases in Japan, separately, using the Japanese version of the EQ-5D-5L^15^. The utilities of asymptomatic chronic hepatitis (ACH), CH, compensated cirrhosis (CC), and decompensated cirrhosis (DC) caused by HB virus or HC virus infection were obtained from outpatients. However, since it is difficult for patients with severe liver conditions, such as HCC, acute hepatitis (AH), and fulminant hepatitis (FH), and patients undergoing post-liver transplantation (PLT) to evaluate their own conditions, in this study, we estimated the utility of these conditions from hepatitis experts.

## Methods

### Subjects

From February 1 to July 31, 2012, we conducted a mail survey and distributed a self-administered questionnaire to 9,952 outpatients with CH, hepatic cirrhosis, or HCC caused by hepatitis B virus (HBV) or HCV infection at 34 medical facilities, including the National Hospital Organization and National Center for Global Health and Medicine in Japan. The collection rate was 63.6%, and 6,331 eligible subjects were included in our study. The survey comprised basic demographic information such as sex, age, disease history, and EQ-5D-5L score.

Patients selected their own disease in the following six medical conditions: (1) CH, (2) hepatic cirrhosis, (3) HCC, (4) viral carrier, (5) fatty liver, and (6) others. Patients with hepatic cirrhosis who had at least one of the following conditions were diagnosed with DC: ascitic fluid, encephalopathy, or albumin level ≤ 3.0 g/dL. None of the patients was diagnosed with CC. Patients who were viral carriers were diagnosed with ACH. When patients selected more than one disease, the diseases were redefined, as shown in Table 1. Patients with HCC and HBV and HCV infections were excluded from this study.

**Table 1:** Disease classification of hepatitis-related conditions.

Redefined condition	Conditions selected by patients
Chronic hepatitis	(1)(4), (1)(5), (1)(4)(5)
Hepatic cirrhosis*	(1)(2), (2)(4), (2)(5), (1)(2)(4), (1)(2)(5), (1)(2)(4)(5)
Hepatocellular carcinoma	(1)(3), (2)(3), (3)(4), (3)(5), (1)(2)(3), (1)(3)(4), (2)(3)(4), (2)(3)(5), (1)(3)(4)(5), (1)(2)(3)(5)
Asymptomatic hepatitis	(4)(5)
Others	(1)(6), (4)(6), (5)(6), (1)(3)(6), (1)(4)(6)

(1) chronic hepatitis, (2) hepatic cirrhosis, (3) hepatocellular carcinoma, (4) viral carrier, (5) fatty liver, and (6) others.

*Patients who had at least one of the following conditions were diagnosed with decompensated cirrhosis: ascitic fluid, encephalopathy, or albumin level ≤ 3.0 g/dL. Others were diagnosed with compensated cirrhosis.

### EuroQol 5-dimension 5-level

The EQ-5D-5L is a self-administered questionnaire in which the respondent records patients’ health states in five dimensions: mobility, self-care ability, ability to undertake usual activities, pain and discomfort, and anxiety and depression. Each dimension is scored on five levels: (1) no problems, (2) slight problems, (3) moderate problems, (4) severe problems, and (5) extreme problems, as shown in Table 2. The health states of each hepatitis condition were converted into a weighted health state index by applying scores from the EQ-5D-5L preference weights elicited from the general population samples. For this study, Japanese population weights were converted to an EQ-5D-5L index score^15^.

**Table 2:** Five dimensions of Health Questionnaire English version (EQ-5D-5L).

1. Mobility					
2. Self-care					
3. Usual activities					
4. Pain/discomfort,					
5. Anxient/depression					
Each dimension has five levels (no problems, slight problems, moderate problems,					
severe problems, unable). The scores of the five dimensions were also converted to					
EQ-5D utilityindex scores					

### The utility estimates by hepatitis experts

A Delphi study was conducted among three experts who engaged in medical care for viral hepatitis over 15 years in a highly advanced medical institution in Japan. First, each of them estimated the utilities of HB- and HC-related diseases, such as AH, FH, ACH, CH, CC, DC, HCC (stage I/II), HCC (stage III/IV), and PLT using the EQ-5D-5L based on a literature review and their experience. Subsequently, a series of panel discussions followed face-to-face at Tokyo University to share each utility estimate and modify the estimates through the Delphi method. The results of ACH, CH, CC, and DC were compared to patient-reported scores, and the results of each condition, including more severe ones, were reviewed.

**Figure 1:**
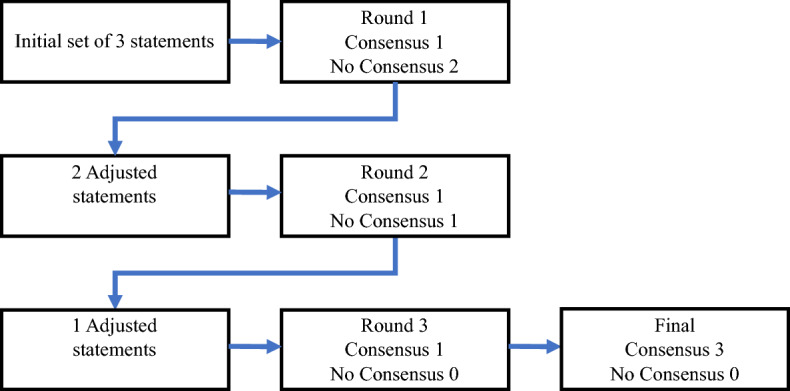
Results per round.

First, each EQ‑5D with five response level statements using a five-point Likert scale was assessed individually. Second, additional feedback was provided by three experts. After each round, the results were analyzed and assessed per statement. Consensus was reached when more than 100% (three experts) of the subjects agreed (strongly agree and agree). When no consensus was reached, the statement was adjusted and presented again in the next round. In these successive rounds, the experts were confronted with de-identified answers from other experts in the previous rounds. After each round, all three experts received a brief summary of the results and were encouraged to give their opinions on the remaining and adjusted statements to reach any consensus on relevant dimensions (Fig. 1).

### Model structure

**Figure 2:**
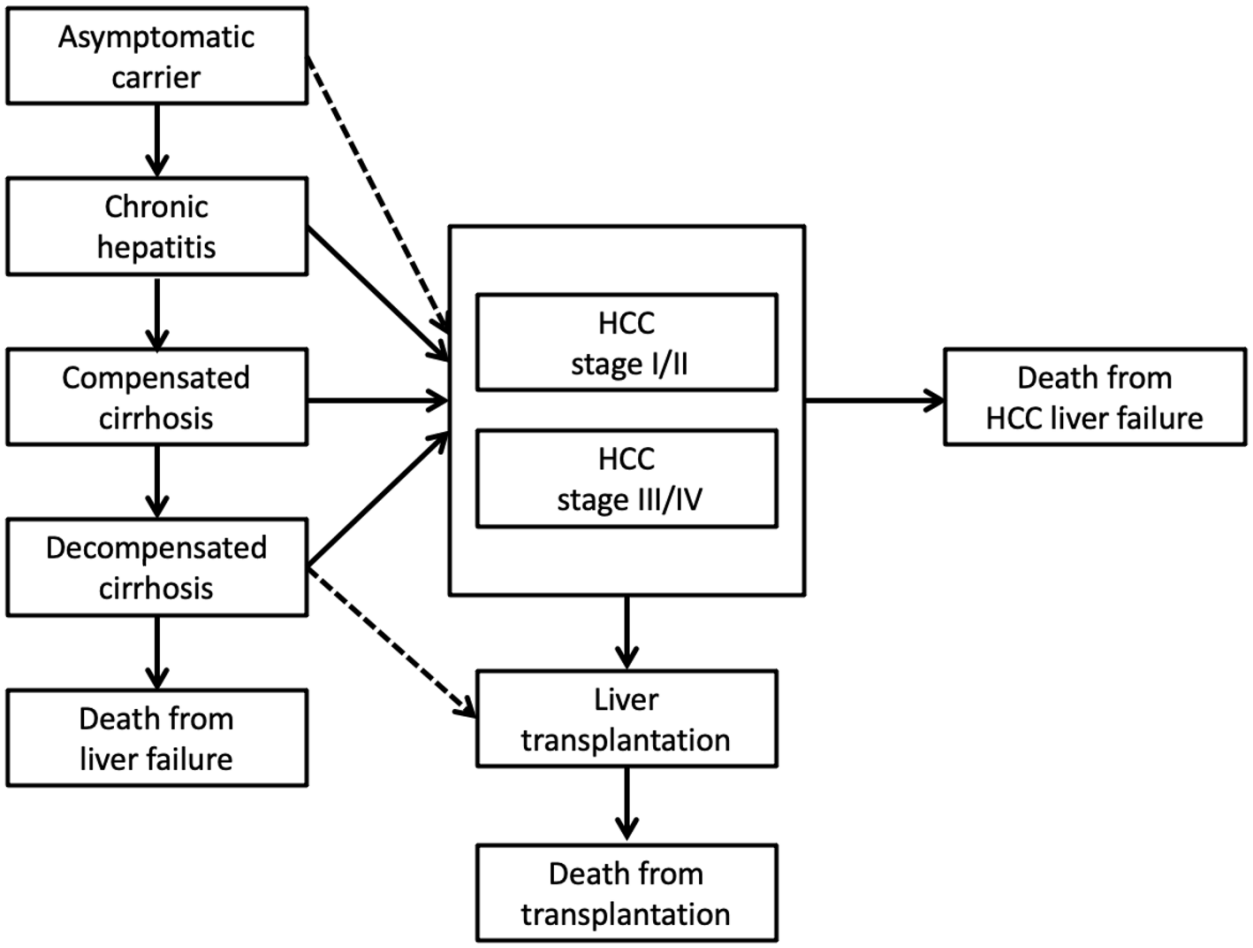
Model structure of chronic hepatitis B-related diseases.

**Figure 3:**
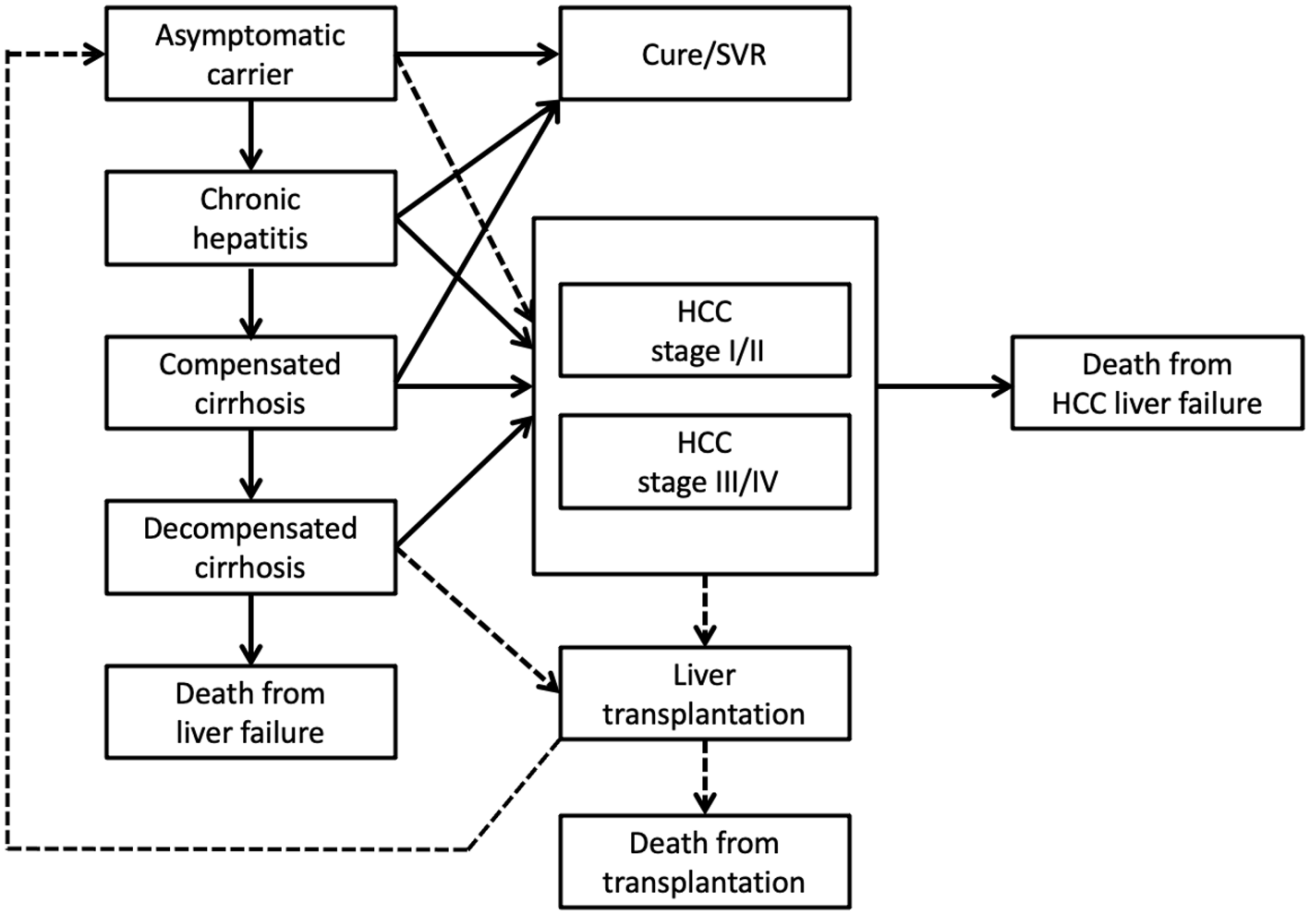
Model structure of chronic hepatitis C-related diseases.

Cost-utility models were constructed separately by virus types, incorporating seven phases in chronic HB, two phases in acute HB, and seven phases in chronic HC, and from asymptomatic carriers toward death, based on prior models of the diseases obtained through systematic literature reviews. The models of chronic HB and HC are described in Figs. 2 and 3, respectively.

### Research ethics approval

The present study was approved by the National Hospital Organization Nagasaki Medical Center (approval number 23065), and the expert-based study was approved by Kagawa University (approval number Heisei24-099). All methods were carried out in accordance with relevant guidelines and regulations regarding research ethics. Furthermore, informed consent was obtained from all subjects and/or their legal guardian(s).

## Result

### Patient-reported utilities

Of the patients (n = 6331) who answered the questionnaire, there were 1322 (721 men and 601 women) eligible subjects with HB and 2875 patients with HC (1263 men and 1612 women). Patient characteristics are shown in Tables 3 and 4.

**Table 3:** Characteristics of patients with hepatitis B.

	Male	Female	Total
ACH	120	157	277
CH	397	347	744
CC	89	52	141
DC	16	19	35
HCC	99	26	125
Total	721	601	1322

*ACH* asymptomatic chronic hepatitis, *CH* chronic hepatitis, *CC* compensated cirrhosis, *DC* decompensated cirrhosis, *HCC* hepatocellular carcinoma.

**Table 4:** Characteristics of patients with hepatitis C.

	Male	Female	Total
ACH	80	125	205
CH	806	1141	1947
CC	110	150	260
DC	43	53	96
HCC	224	143	367
Total	1263	1612	2875

*ACH* asymptomatic chronic hepatitis, *CH* chronic hepatitis, *CC* compensated cirrhosis, *DC* decompensated cirrhosis, *HCC* hepatocellular carcinoma.

The utility scores of HB- and HC-related liver diseases, such as ACH, CH, CC, and DC, are shown in Table 5. In patients with chronic HB, the utilities of ACH, CH, CC, and DC were 0.904, 0.868, 0.845, and 0.722, respectively. However, in patients with chronic HC, the utilities under the same conditions were 0.876, 0.821, 0.737, and 0.671, respectively.

**Table 5:** Utilities of hepatitis-related conditions (All).

Utility	All ages			Under 40 years			40s			50s		
	n	Mean	SD	n	Mean	SD	n	Mean	SD	n	Mean	SD
**Hepatitis B (acute)**												
AH*		0.529										
FH*		− 0.111										
**Hepatitis B (chronic)**												
ACH**	278	0.904	0.133	33	0.933	0.098	44	0.934	0.096	69	0.915	0.131
CH**	745	0.868	0.145	113	0.883	0.132	135	0.893	0.128	186	0.880	0.137
CC**	141	0.845	0.158	4	0.812	0.131	10	0.862	0.102	30	0.873	0.167
DC**	35	0.722	0.22	0			2	0.364	0.368	6	0.915	0.094
HCC (stage I/II)*		0.675										
HCC (stage III/IV)*		0.428										
PLT*		0.651										
**Hepatitis C**												
ACH**	205	0.876	0.154	8	0.919	0.114	18	0.915	0.111	32	0.916	0.127
CH**	1951	0.821	0.176	43	0.834	0.146	114	0.816	0.189	316	0.844	0.153
CC**	260	0.737	0.195	2	0.548	0.062	9	0.688	0.110	30	0.731	0.221
DC**	96	0.671	0.224	1	0.740	–	9	0.801	0.140	10	0.713	0.193
HCC (stage I/II)*		0.675										
HCC (stage III/IV)*		0.428										
PLT*		0.651										

Utility	60s			70s			80s years and over					
	n	Mean	SD	n	Mean	SD	N	Mean	SD			
**Hepatitis B (acute)**												
AH*												
FH*												
**Hepatitis B (chronic)**												
ACH**	94	0.884	0.144	34	0.889	0.152	4	0.759	0.195			
CH**	224	0.859	0.143	73	0.824	0.177	14	0.714	0.217			
CC**	67	0.838	0.159	24	0.833	0.195	6	0.823	0.195			
DC**	13	0.698	0.171	10	0.746	0.244	4	0.628	0.124			
HCC (stage I/II)*												
HCC (stage III/IV)*												
PLT*												
**Hepatitis C**												
ACH**	62	0.864	0.137	73	0.863	0.172	12	0.822	0.234			
CH**	660	0.840	0.159	663	0.802	0.193	155	0.771	0.185			
CC**	85	0.774	0.179	106	0.727	0.201	28	0.700	0.163			
DC*	29	0.665	0.262	40	0.681	0.189	10	0.526	0.257			
HCC (stage I/II)*												
HCC (stage III/IV)*												
PLT*												

*Final consensus value of expert-reported EQ-5D-5L.

**Mean of patient-reported EQ-5D-5L.

*AH* acute hepatitis, *FH* fulminant hepatitis, *ACH* asymptomatic chronic hepatitis, *CH* chronic hepatitis, *CC* compensated cirrhosis, *DC* decompensated cirrhosis, *HCC* hepatocellular carcinoma *PLT* post-liver transplantation.

### Hepatitis expert-reported utilities

The utilities of the HB- and HC-related liver diseases, such as AH, FH, HCC, and PLT, were estimated by three hepatitis experts and were averaged out. In patients with acute HB, the utilities of AH and FH were 0.529 and − 0.111, respectively. In patients with chronic HB and HC, the utilities of HCC (stage I/II), HCC (stage III/IV), and PLT were 0.675, 0.428, and 0.651, respectively. The utilities reported by the patients and those estimated by the experts are integrated in Table 5 (All), Table 6 (Male), and Table 7 (Female).

**Table 6:** Utilities of hepatitis-related conditions (Male).

Utility	All ages			Under 40 years			40s			50s		
	n	Mean	SD	n	Mean	SD	n	Mean	SD	n	Mean	SD
**Hepatitis B (acute)**												
AH*												
FH*												
**Hepatitis B (chronic)**												
ACH**	120	0.920	0.122	11	0.934	0.096	19	0.942	0.106	29	0.937	0.107
CH**	397	0.884	0.139	61	0.877	0.148	73	0.898	0.125	109	0.898	0.134
CC**	89	0.858	0.149	4	0.812	0.131	9	0.866	0.107	24	0.868	0.164
DC**	16	0.756	0.195	0	–	–	1	0.624	0.368	3	0.943	0.099
HCC (stage I/II)*		0.675										
HCC (stage III/IV)*		0.428										
PLT*		0.651										
**Hepatitis C**												
ACH**	80	0.891	0.148	3	0.785	0.045	8	0.948	0.097	10	0.915	0.150
CH**	806	0.842	0.170	19	0.847	0.145	62	0.794	0.196	140	0.858	0.151
CC**	110	0.782	0.188	1	0.592	–	5	0.656	0.135	17	0.787	0.195
DC**	43	0.721	0.181	0	–	–	4	0.748	0.125	6	0.782	0.168
HCC (stage I/II)*		0.675										
HCC (stage III/IV)*		0.428										
PLT*		0.651										

Utility	60s			70s			80s years and over					
	n	Mean	SD	n	Mean	SD	n	Mean	SD			
**Hepatitis B (acute)**												
AH*												
FH*												
**Hepatitis B (chronic)**												
ACH**	40	0.905	0.125	20	0.929	0.133	4	0.759	0.195			
CH**	112	0.874	0.133	36	0.885	0.142	6	0.716	0.268			
CC**	37	0.861	0.147	13	0.816	0.164	2	1.000	0.000			
DC**	7	0.710	0.228	4	0.773	0.154	1	0.583				
HCC (stage I/II)*												
HCC (stage III/IV)*												
PLT*												
**Hepatitis C**												
ACH**	23	0.873	0.115	28	0.889	0.191	8	0.900	0.115			
CH**	245	0.855	0.153	272	0.832	0.187	68	0.846	0.173			
CC**	35	0.806	0.140	44	0.795	0.186	8	0.694	0.217			
DC**	13	0.712	0.184	13	0.738	0.181	2	0.404	0.025			
HCC (stage I/II)*												
HCC (stage III/IV)*												
PLT*												

*Final consensus value of expert-reported EQ-5D-5L.

**Mean of patient-reported EQ-5D-5L.

*AH* acute hepatitis, *FH* fulminant hepatitis, *ACH* asymptomatic chronic hepatitis, *CH* chronic hepatitis, *CC* compensated cirrhosis, *DC* decompensated cirrhosis, *HCC* hepatocellular carcinoma *PLT* post-liver transplantation.

**Table 7:** Utilities of hepatitis-related conditions (Female).

Utility	All ages			Under 40 years			40s			50s		
	n	Mean	SD	n	Mean	SD	n	Mean	SD	n	Mean	SD
**Hepatitis B (acute)**												
AH*		0.529										
FH*		− 0.111										
**Hepatitis B (chronic)**												
ACH**	157	0.892	0.140	22	0.933	0.103	43	0.902	0.143	53	0.867	0.157
CH**	347	0.849	0.149	52	0.889	0.111	77	0.855	0.139	112	0.844	0.152
CC**	52	0.823	0.173	0	–	–	6	0.893	0.195	30	0.811	0.171
DC**	19	0.693	0.239	0	–	–	3	0.886	0.099	6	0.685	0.085
HCC (stage I/II)*		0.675										
HCC (stage III/IV)*		0.428										
PLT*		0.651										
**Hepatitis C**												
ACH**	205	0.876	0.154	5	0.919	0.000	10	0.889	0.119	22	0.917	0.120
CH**	1951	0.821	0.176	24	0.834	0.150	52	0.843	0.177	176	0.834	0.155
CC**	260	0.737	0.195	1	0.548	–	4	0.729	0.064	13	0.658	0.238
DC**	96	0.671	0.224	1	0.740	–	2	0.907	0.132	4	0.610	0.203
HCC (stage I/II)*		0.675										
HCC (stage III/IV)*		0.428										
PLT*		0.651										

Utility	60s			70s			80s years and over					
	n	Mean	SD	n	Mean	SD	n	Mean	SD			
**Hepatitis B (acute)**												
AH*												
FH*												
**Hepatitis B (chronic)**												
ACH**	53	0.867	0.157	14	0.833	0.164	0	–	–			
CH**	112	0.844	0.152	36	0.759	0.187	8	0.713	0.191			
CC**	30	0.811	0.171	11	0.851	0.178	4	0.734	0.178			
DC**	6	0.685	0.085	6	0.727	0.303	3	0.643	0.148			
HCC (stage I/II)*												
HCC (stage III/IV)*												
PLT*												
**Hepatitis C**												
ACH**	39	0.859	0.150	45	0.848	0.160	4	0.664	0.347			
CH**	413	0.832	0.162	390	0.780	0.195	86	0.712	0.175			
CC**	50	0.752	0.201	62	0.679	0.199	20	0.705	0.143			
DC**	16	0.626	0.313	22	0.635	0.187	8	0.557	0.282			
HCC (stage I/II)*												
HCC (stage III/IV)*												
PLT*												

*Final consensus value of expert-reported EQ-5D-5L.

**Mean of patient-reported EQ-5D-5L.

*AH* acute hepatitis, *FH* fulminant hepatitis, *ACH* asymptomatic chronic hepatitis, *CH* chronic hepatitis, *CC* compensated cirrhosis, *DC* decompensated cirrhosis, *HCC* hepatocellular carcinoma *PLT* post-liver transplantation.

## Discussion

This study showed the utilities of both HB- and HC-related diseases in a large-scale Japanese population using the EQ-5D-5L. This study suggests that QOL is markedly diminished in HCB and HCV patients, and the utilities of HB-related diseases were slightly higher than those of HC-related diseases, comparably disease conditions. These data are similar to those of previous studies^10,16^.

Other studies have reported similar results. For example, Chong et al.^16^ have shown that the utilities of moderate HC (n = 44), HC-related CC (n = 24), DC (n = 9), HCC (n = 15), and PLT (n = 30) in the Canadian population using the EQ-5D-3L were 0.76, 0.74, 0.66, 0.65, and 0.69, respectively. These data for DC, HCC, and PLT were similar to those in our study. The patients with HCC in this study were considered to have stage I or II HCC because they were only outpatients. The data for moderate HC and CC were slightly lower than those in our study. This may be because the utilities in the Canadian population were measured using the EuroQol 5-Dimension 3-Level (EQ-5D-3L), but those in our study were measured using the EQ-5D-5L^17^. Moreover, Saeed et al. have recently reported a meta-analysis of health utilities in patients with chronic HC. These utilities were mild/moderate CH (0.751), CC (0.671), HCC (0.662), and DC (0.602)^18^. Their utility data also seem to be slightly lower than those in our study. In a clinical setting involving patients with CH, a relative 7% reduction in ”the ceiling effect” was found in the EQ-5D-5L from that of the EQ-5D-3L, and the EQ-5D-5L was feasible and had promising levels of performance^17^.

In contrast, Woo et al.^10^ have shown that the utilities of non-cirrhotic HB (n = 294), HB-related CC (n = 79), DC (n = 7), HCC (n = 23), and PLT (n = 30) in the Canadian population using the EQ-5D were 0.92, 0.88, 0.73, 0.81, and 0.84, respectively. Moreover, Ong et al.^19 ^ have shown that the utilities of each condition were similar to or slightly higher than Woo’s data. In these studies, the utilities of HCC and PLT were higher than those in our study. This may be because the patients in the studies were different from our patients in that their patients were only outpatients and their symptoms might be more moderate.

This study was conducted with reference to the two guidelines of the NICE^14,20^. These two guidelines recommend that the EQ-5D is the preferred measure of HRQoL in adults. The EQ-5D probably remains the preferred questionnaire on the following grounds: (1) it meets the reference case criteria; (2) it is the most widely used generic preference-based measure; thus, more studies will prefer using this questionnaire; (3) it has been shown to have acceptable psychometric properties across a wide range of common conditions (including rheumatoid arthritis, hip fracture, intermittent claudication, liver transplantation); (4) there is no other generic preference-based measure that is likely to displace this position in the near future; and (5) for the sake of continuity with previous technology appraisals, it is better to use the EQ-5D unless a significantly better generic measure is determined.

The NICE guidelines state that HRQoL, or changes in HRQoL, should be measured directly by patients. Moreover, the utilities estimated by experts are known to be significantly different from those reported by patients^16,21–23^. It has been reported that quality of life indices tend to be overly valued by clinicians^22^. However, some authors have described that the proxy could assess the patient’s mobility and self-care accurately^24^ and moderate agreement between responses from patients and those from their proxies for at least the observable domains of the EQ-5D^24,25^. In general, some domains of EQ-5D obtained from a proxy may be sufficiently valid and unbiased for use in most types of trials and surveys^24^. In another study^26^, the total EQ-5D score was significantly positively correlated between patients at the end stage of lung cancer reporting and nurses reporting. Moreover, the scores for some items, including “mobility,” “self-care,” and “pain/discomfort” of EQ-5D, have statistically significant positive correlation between patients and nurses^26^. In terms of the Chronic Liver Disease Questionnaire (CLDQ), a liver disease-specific instrument used for the assessment of HRQoL, since the HRQOL of patients with HCC is greatly altered both qualitatively and quantitatively, they were not included in the study that developed CLDQ^26,27^.

Several limitations of this study should be acknowledged. First, ethics and privacy regulations precluded the addition of region-specific utilities. However, this national data from patient-reported EQ-5D-5L was collected from 34 medical facilities, including the National Hospital Organization and National Center for Global Health and Medicine in Japan across the country (which are located from the northern region of Japan: Hokkaido to the southern region of Japan: Kyushu of Japan) that hepatitis specialists affiliated with, and hepatitis patients receive the standardized specialized treatments from the guideline. Second, because of the very limited number of patients and the low probability of being able to ask questions, the QOL in AH and FH in acute hepatitis B and HCC and PLT in chronic hepatitis B and C was obtained by expert agreement. The EQ5D questionnaire was completed on the assumption of a standard condition in these diseases. When responding, the intensity of symptoms was considered to some extent, but the fact that the fluctuation of symptoms was not sufficiently captured is considered to be one of the limitations. However, this study was not based on the focus of QOL values of individual conditions such as AH, HF, HCC and PLT, but was conducted to construct a natural history model of hepatitis and utilize it for cost-effective analysis and estimated the national disease burden studies. Therefore, the individual health state was not evaluated in detail. Third, the human QOL is evaluated including the complication (comorbidity) in the last stage of hepatitis. Therefore, QOL adjusted for complications is not the purpose of this study. However, further study is required.

In this study, data from more severe conditions were estimated by three hepatitis experts. Because our study mainly comprised outpatients, data of patients with severe hepatitis who were largely admitted to hospitals were biased, and these patients may have difficulty in evaluating their own conditions^25^. Certainly, the utility of HCC reported by outpatients in our study was relatively higher than that reported in other studies^16,28^, because their symptoms were relatively moderate. Subsequently, the utilities of more severe hepatitis-related diseases, such as HCC, AH, FH, and PLT, were estimated by hepatitis experts. Moreover, since there is the potential for patients not to recognize their own conditions correctly, in a study based on a self-administered questionnaire, disease conditions might be misclassified^29^. Furthermore, hepatitis expert-reported utilities in less severe conditions, such as ACH, CH, CC, and DC, were similar to those of patient-reported utilities under the same conditions. This might suggest that our results from hepatitis expert-reported utilities in severe conditions such as HCC (stage I/II), HCC (stage III/IV), PLT, AH, and FH were reasonable and reliable. However, the value judgment of the utilities requires further investigation.

## Data Availability

The datasets generated and/or analyzed during the current study are not publicly available due to “the IRB in Kagawa University” but are available from the corresponding author on reasonable request.
